# Prediction Models for Radiological Characterization of Natural Aggregates Based on Chemical Composition and Mineralogy

**DOI:** 10.3390/ma18061369

**Published:** 2025-03-20

**Authors:** Andrés Caño, María del Mar Alonso, Alicia Pachón-Montaño, Queralt Marzal, Guillermo Hernáiz, Luís Sousa, José Antonio Suárez-Navarro

**Affiliations:** 1 Eduardo Torroja Institute for Construction Sciences (IETcc-CSIC), 28033 Madrid, Spain; andres.cano@ietcc.csic.es (A.C.); apachon@ietcc.csic.es (A.P.-M.); queraltbelen.marzal@ietcc.csic.es (Q.M.); 2 Environmental Radioactivity and Radiological Monitoring Unit (URAyVR), CIEMAT, Avda Complutense, 40, 28040 Madrid, Spain; guillermo.hernaiz@ciemat.es (G.H.); ja.suarez@ciemat.es (J.A.S.-N.); 3 Department of Geology and Pole of Geosciences Center, University of Trás-os-Montes e Alto Douro, 5000-801 Vila Real, Portugal; lsousa@utad.pt

**Keywords:** ACI, ACP, aggregates, gamma spectrometry, XRF, NORM

## Abstract

The radiological characterization of aggregates used in construction materials is essential to determine their suitability from a radiological protection perspective and to ensure their safety for health and the environment. While the activity concentrations of radionuclides present in construction materials are typically determined using gamma spectrometry, an alternative approach involves the development of statistical methods and predictive models derived from the chemical composition of the material. A total of 39 aggregates used in construction of various types (siliceous, carbonatic, volcanic, and granitic) have been analyzed, correlating their chemical compositions obtained through X-ray fluorescence (XRF) with the activity concentrations of natural radionuclides measured via gamma spectrometry using principal component analysis (PCA). The results obtained allowed for the observation of an inversely proportional relationship between the chemical composition of the grouping of siliceous and carbonatic aggregates and the content of radionuclides. However, the set of granitic aggregates showed a strong correlation with the natural radioactive series of uranium, thorium, and ^40^K. Conversely, the radionuclide content of volcanic aggregates was independent of their chemical composition. The results obtained from the PCA facilitated the development of different models using multiple regression analysis. The chemical parameters obtained in the proposed models were related to the typical mineralogy in each grouping, ranging from primary minerals such as feldspars to accessory minerals such as anatase, apatite, and pyrolusite. Finally, the models were validated using independent samples from those used to determine the models, achieving RSD (%) values ≤ 30% in 50% of the activity concentrations of ^226^Ra, ^232^Th(^212^Pb), and ^40^K, as well as the estimated ACI.

## 1. Introduction

The aggregates used in concrete production can have different morphologies (rounded or crushed) and varied chemical compositions depending on their nature or origin, which is often determined by their geographical availability. Additionally, they can originate from the recycling of construction materials [1,2,3] or have a natural source. Natural aggregates can be classified based on their chemical and mineralogical composition as siliceous, carbonaceous, volcanic, and granitic. The choice of one type of aggregate or another (or their combination) as a construction material has a significant influence on the physical–mechanical properties, rheology, and durability of concrete [4,5,6,7,8]. However, a generally overlooked issue is the content of natural radionuclides, whose excess above the environmental radiological background can pose risks to human health and the environment. The content of natural radionuclides in aggregates is related to their geological origin and mineralogy. In this regard, granites often contain high activity concentrations of natural radionuclides, primarily due to the presence of accessory mineral phases such as allanite, monazite, zircon, apatite, chevkinite, and riebeckite, which are usually enriched in U and Th [9]. However, these rocks can exhibit variable activity concentrations in different radionuclides depending on their origins and petrography [9,10,11]. In fact, the high presence of radionuclides from the ^238^U series in granites is the cause of elevated emissions of ^222^Rn, with harmful health effects in constructions using this type of rock [12,13,14].

In the case of volcanic rocks, they can also exhibit high content of radioactive elements. The concentration of radioactive elements in volcanic rocks often depends on the geological context and the volcanic processes that generate them [15]. Similarly to granitic rocks, their use as construction materials can pose risks to health and the environment [16,17,18].

The presence of higher radioactive content in construction materials led to the introduction of the Activity Concentration Index (ACI) in the European Directive EURATOM 2013/59. The ACI is used as a screening tool to assess the suitability of a construction material from a radiological protection perspective [19]. The ACI is calculated based on the activity concentrations of the following radionuclides: ^226^Ra, ^232^Th, and ^40^K. These radionuclides are typically measured using gamma spectrometry, a non-destructive analysis method that also allows for the simultaneous measurement of several natural radionuclides [20,21]. However, gamma spectrometry is not a routine analysis method and requires expensive detectors, as well as qualified personnel, to perform these determinations. Therefore, there is a need to explore alternative methodologies capable of simplifying the characterization of the radionuclide content in construction materials.

Currently, the existing literature includes research that examines the relationship between the activity concentrations of natural radionuclides and mineralogy in soils, sediments, and rocks, with potential applications as construction materials. These studies have determined that the radiological risk is influenced not only by the possible presence of clays but also by certain minerals enriched in radionuclides, such as zircon, monazite, and other accessory minerals [22,23,24].

Previous studies have employed methodologies based on principal component analysis (PCA) to correlate physicochemical parameters with the content of natural radionuclides in various construction materials. Some researchers have used this statistical tool to determine the chemical factors affecting radon emanation in ceramic materials [25], to correlate the activity concentration with the particle size of Fe_2_SiO_4_ used as a substitute for aggregates in concretes and mortars [26], or even to evaluate the suitability of certain granitic rocks by correlating the activity concentrations of their natural radionuclides with different radiological protection indices [27].

In a recent study, the correlation between the chemical composition and the activity concentration of natural radionuclides in cements and supplementary cementitious materials (SCMs), such as fly ash and slags from the metallurgical industry, was investigated [28], enabling the development of predictive models for ^226^Ra, ^232^Th(^212^Pb), and ^40^K.

However, there is a lack of research proposing estimation models based on the chemical composition of natural aggregates that could be used to estimate the activity concentrations of natural radionuclides. For this reason, the purpose of this work was to investigate whether there is a possible correlation between the chemical composition, obtained through X-ray fluorescence (XRF), and the content of radionuclides, obtained through gamma spectrometry, in different types of natural aggregates using principal component analysis (PCA). The types of aggregates included in this study were those commonly used in concrete production, namely siliceous, carbonate, volcanic, and granitic aggregates [9,22,29,30,31,32,33]. On the other hand, although the use of mixed aggregates is common in concrete preparation, this work studied the aggregates independently to obtain correlations that could subsequently be scaled. The new models to be investigated are intended to continue this type of research across different types of aggregates so that, in combination with those developed for cementitious materials, a tool is available to design concretes that do not pose a risk to health and the environment.

## 2. Materials and Methods

### 2.1. Materials

**Figure 1:**
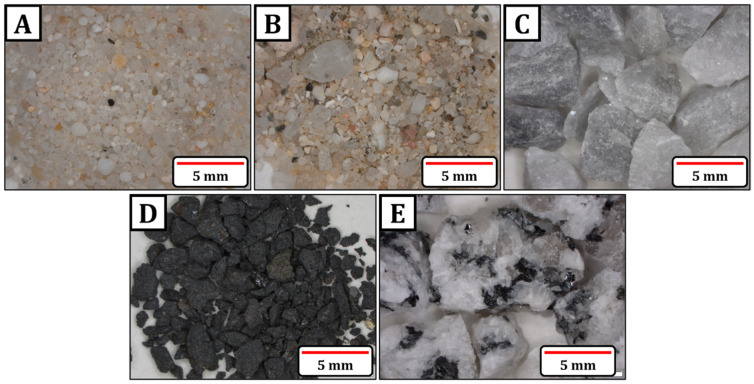
Morphological characteristics of aggregates from the four groups analyzed in this study: (**A**) quartzitic siliceous aggregate (S12), (**B**) feldspathic (S9), (**C**) carbonate aggregate (C2), (**D**) volcanic aggregate (V3), and (**E**) granitic aggregate (G6).

In this study, 39 aggregates of different geological origins and chemical compositions, widely used in concrete preparation, were analyzed. The aggregates were classified into four types: siliceous aggregates (S), carbonate aggregates (C), volcanic aggregates (V), and granitic aggregates (G). Figure 1 shows an image of each type of aggregate studied. The designations of the different aggregates were as follows.

- S: 8 siliceous gravels and sands of quartzitic composition (S1, S3, S5, S6, S7, S10, S11, and S12) and 4 feldspathic ones (S2, S4, S8, and S9).
- C: 5 carbonates, with a calcitic composition (C3 and C5) and dolomitic composition (C1, C2, and C4).
- V: 7 rocks of volcanic origin (V1–V7).
- G: 15 gravels and sands of granitic origin (G1–G15).

### 2.2. Methods

**Figure 2:**
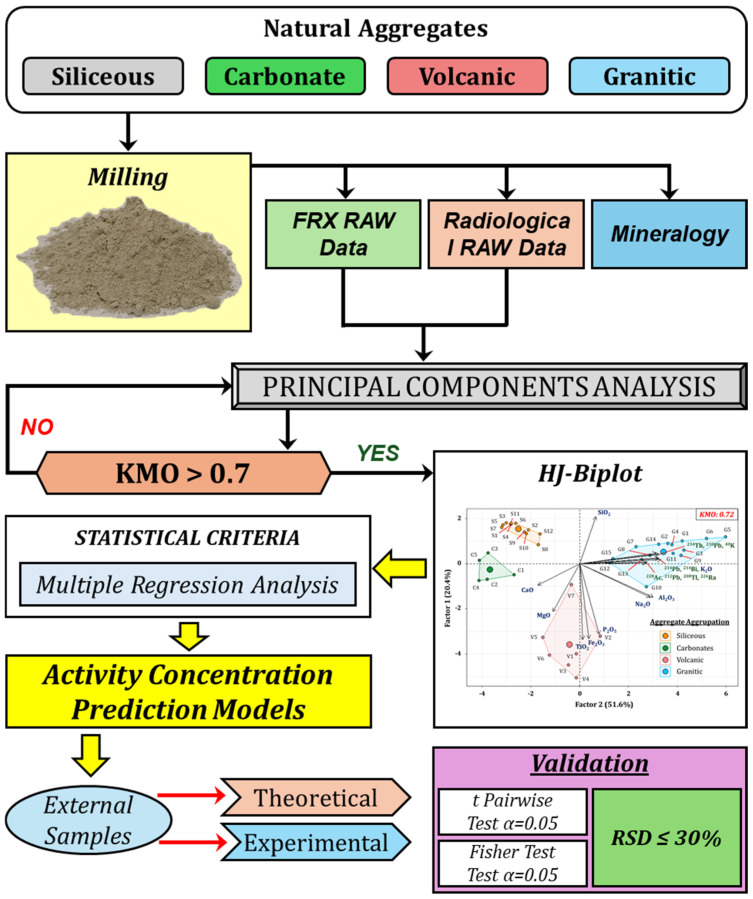
Flowchart summarizing the methodology used in this study.

Figure 2 shows a flowchart summarizing the main aspects of the research conducted. The four types of aggregates selected in this study—siliceous, carbonaceous, volcanic, and granitic—were ground and sieved to a particle size of 20 μm. Subsequently, the chemical composition (XRF), the mineralogical composition (XRD), and the natural radionuclides present were determined using gamma spectrometry. The results obtained through XRF and gamma spectrometry were employed to conduct a principal component analysis (PCA). The criterion for the application of PCA was to have a number of samples greater than the number of variables involved in the analysis (percentage of oxides and activity concentrations of natural radionuclides) and to obtain a Kaiser–Meyer–Olkin (KMO) value greater than 0.7 to ensure adequate correlation. The PCA was represented using an HJ-Biplot graph. Subsequently, a multiple regression analysis (MRA) was applied, considering the clusters obtained through PCA. The variables used in the fitting were initially filtered by applying a collinearity test, eliminating those that showed a variance inflation factor (VIF) greater than 10. The resulting variables were adjusted using the forward and backward elimination methods. The mathematical models obtained through MRA were used to determine the activity concentration index (ACI) using a number of samples corresponding to 10% of the total samples for each aggregate. The validity criterion used was an RSD (%) lower than 30%. Additionally, the means and variances were compared using Student’s *t*-test and Fisher’s F-test.

#### 2.2.1. Chemical and Mineralogical Compositions of the Aggregates

The chemical compositions of the 39 aggregates analyzed were determined using X-ray fluorescence (XRF) on a Bruker S8 Tiger analyzer (Bruker, Billerica, MA, USA). The loss on ignition (LoI) was obtained according to the EN 196-2:2014 standard [34]. The chemical compositions of six aggregates included in this study (S1, V1, V2, and G1–G3) were extracted from a previous work [35].

The mineralogical compositions of the aggregates were determined using X-ray diffraction (XRD) with a Bruker-AXS D8 Advance diffractometer (Bruker, Billerica, MA, USA) employing Cu-Kα_1_ radiation over a 2θ range of 5–70°. Two samples of each type of aggregate were selected and ground in an XRD-Mill McCrone (Retsch, Haan, Germany) to achieve a particle size of less than 20 µm. The identification of the crystalline phases present was performed using the Diffrac.EVA V4.2 software (Bruker, Billerica, MA, USA) and the Crystallography Open Database (COD). The quantitative analysis of the mineral phases in the samples was conducted using the Rietveld method with the TOPAS V5 software (Bruker AXS). To quantify the percentage of amorphous phases present in the granitic-origin aggregates, the internal standard method was used. Here, 20% corundum addition was introduced, which was obtained from the synthetic compound α-Al_2_O_3_ 99.9% (Thermo Scientific, Waltham, MA, USA), previously calcined at 1600 °C for 1 h, and subsequently milled to below 20 microns, with the particle size of the sample with which it was mixed. The structural and crystallographic parameters of the identified phases were refined to generate a theoretical diffraction pattern. The quality of the fit between the theoretical pattern and the experimental data was evaluated using the R_wp_ index.

#### 2.2.2. Gamma Spectrometry

The activity concentrations of radionuclides present in the various analyzed aggregates were determined using gamma spectrometry at the URAyVR laboratory of CIEMAT, accredited by ENAC, in accordance with the ISO/IEC 17025:2017 standard [36].

**Figure 3:**
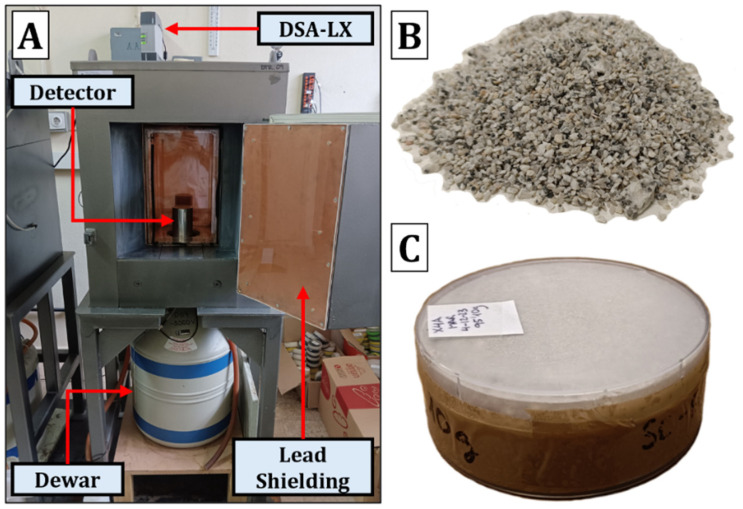
(**A**) Schematic of the gamma spectrometry equipment used; (**B**) granitic sand before being ground; and (**C**) ground sample sealed in the cylindrical geometry container used for sample measurement.

Measurements were conducted using four coaxial HPGe detectors from Canberra Industries: 2 extended-range (XtRa), 1 broad-energy (BeGE), and 1 reverse-electrode type (ReGE). Figure 3 shows a diagram of the high-purity germanium (HPGe) detector used in this research. The detector was cooled using a dewar container filled with liquid nitrogen. It was housed within passive shielding composed of Fe and Pb, which was 15 cm thick. The shielding included two layers of Cu and Zn, each 1.5 mm thick, to absorb the X-rays emitted by the Pb. The detectors were connected to a DSA-LX module (Mirion Canberra, Atlanta, GA, USA) that integrated all necessary electronics: a high-voltage power supply, amplifier, analog-to-digital converter, and communication module with a computer. The spectra were acquired and analyzed using the Genie 2000 software (version 3.4.1) [37]. Efficiency curves as a function of energy were calculated using the LabSOCS software (version 4.4.1), as the four detectors used were characterized by Canberra [20]. The samples were measured for 80,000 s, while the backgrounds were measured for 600,000 s. Quality control of the equipment was performed monthly. Additionally, the laboratory participated in at least two national and international intercomparison exercises.

The samples were prepared using the following procedure: (i) drying at 105 °C for 24 h until a constant weight was achieved, (ii) grinding using a disc mill (Retsch RS200, Retsch, Haan, Germany), (iii) sieving to obtain a particle size of 63 μm, (iv) placing the sample in a cylindrical polypropylene box with a diameter of 76 mm and a height of 30 mm, and (v) sealing the box with parafilm to prevent losses of ^222^Rn. The samples were allowed to stand for 21 days to reach a secular equilibrium between ^226^Ra and its short-lived gamma-emitting descendants (^214^Pb and ^214^Bi). The activity concentration of ^226^Ra was determined directly from the 186 keV peak, suppressing interference from ^235^U using the method described in [38].

Table 1 summarizes the energies and photon yields per 100 disintegrations (%) of the radionuclides included in this study, which belonged to the natural radioactive decay series of uranium (^234^Th, ^226^Ra, ^214^Pb, ^214^Bi, and ^210^Pb) and thorium (^228^Ac, ^212^Pb, and ^208^Tl), in addition to ^40^K [39].

#### 2.2.3. Data Analysis

The percentages of the oxides determined by X-ray fluorescence (XRF) were correlated with the activity concentrations of the natural radionuclides using principal component analysis (PCA). The PCA was conducted with the RStudio statistical software (version 2004.09.0 Build 375) using the Factoextra and FactoMineR libraries [40]. PCA allowed for the grouping of different aggregates and the correlation of various variables (oxide percentages and activity concentrations) based on their variances. The correlations between the variables and the sample scores were represented using an HJ-Biplot [28]. The correlations among the different variables were determined by the cosine of the angle that they formed with each other. Furthermore, the relationship among all of the variables collectively within the PCA was quantified using the Kaiser–Meyer–Olkin (KMO) suitability index [41]. The different variables were adjusted using multiple regression analysis, taking into account the groupings obtained in the PCA, using the RStudio statistical software. The selection of variables was based on the following criteria:

1. Variables with a percentage of values above the limit of detection (LoD) of less than 25% were removed to prevent zero-inflated data, which could potentially introduce biases that distort the model [42];
2. Variables with a variance inflation factor (VIF) value above 10 were iteratively removed to avoid collinearity among the variables [43,44];
3. Variables whose variance exceeded the significance level of 0.05 were eliminated when applying backward stepwise regression, as they were not statistically significant.

On the other hand, the influence of the final variables of the models was assessed by standardizing the coefficients obtained in the multiple regression analysis. The standardization of the coefficients was carried out using the standard deviation of the explanatory variables. The variables with the greatest weight in the adjusted models were those with the highest absolute standardized coefficient values.

Finally, the accuracy of the models was assessed using the root mean square error (RMSE), considering the experimentally obtained activity concentrations as observed values, compared to those obtained through the application of the proposed models as estimated values. RMSE values close to 0 indicated greater accuracy of the obtained model.

#### 2.2.4. Validation of the Models for the Estimation of the Activity Concentrations of ^226^Ra, ^232^Th (^212^Pb), and ^40^K

The accuracy of the obtained models was assessed using external samples of aggregates with typologies equivalent to those used to construct the models. The numbers of samples used for validation were as follows: 1 siliceous aggregate, 1 carbonated aggregate, and 2 granitic aggregates, corresponding to 12.5%, 20.0%, and 13.3%, respectively. These numbers were considered adequate for the set of samples used and are the standard values employed in the quality control of samples of radiological origin [45]. The models were validated based on the activity concentration index (ACI), which is determined using the expression in Equation (1) [19]:

(1) $$ACI=\frac{{\;C}^{226}Ra}{300}+\frac{{\;C}^{232}Th}{200}+\frac{{\;C}^{40}K}{3000}$$

where $C^{226}Ra$ is the activity concentration of ^226^Ra, $C^{232}Th$ is the activity concentration of ^232^Th (indirectly obtained using the activity concentration of ^212^Pb), and $C^{40}K$ is the activity concentration of ^40^K.

The results obtained through the application of the models to the validation samples were compared with experimental results obtained via gamma spectrometry using the relative standard deviation (RSD (%)). The acceptance criterion for the models was based on previous studies [28]—specifically, RSD (%) ≤ 30%.

## 3. Results and Discussion

### 3.1. Chemical Composition and Activity Concentrations of Natural Radionuclides in Aggregates

The results obtained through XRF and the activity concentration values of the natural radionuclides in the analyzed aggregates are presented in Tables S1 and S2 of the Supplementary Materials, respectively.

**Figure 4:**
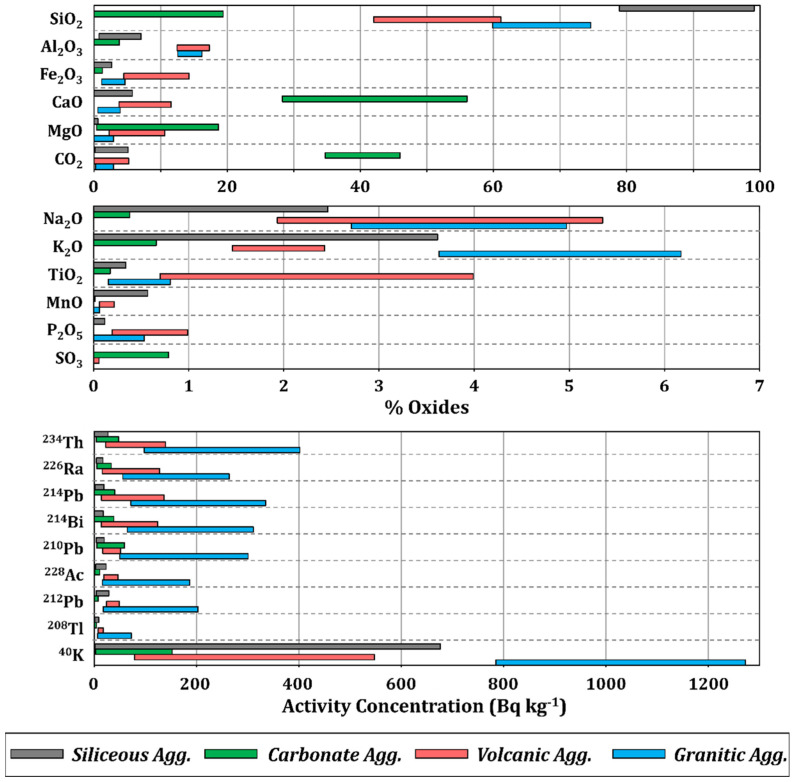
Chemical composition (wt.%) and activity concentrations (Bq kg^−1^) of the radionuclides from the natural uranium and thorium radioactive series, along with ^40^K, for the set of aggregates included in this study.

Figure 4 shows the ranges of the oxide percentages obtained through XRF and the activity concentrations of the radionuclides determined via gamma spectrometry for the set of aggregates considered in this study. Siliceous aggregates are shown in a gray color, carbonates in green, volcanic aggregates in red, and granitic ones in blue. The percentages of oxides determined were represented in two ranges: 0–100% and 0–7%.

The chemical composition of the silica aggregates reflects a SiO_2_ percentage between 78.9 and 99.2%, which explains the predominance of quartz as the mineral phase in this group of aggregates. Additionally, a lower percentage of Al_2_O_3_ (0.8–7.1%), CaO (0.0–5.8%), K_2_O (0.0–3.6%), and Na_2_O (0.0–2.5%) is observed. The loss on ignition (0.1–5%) may indicate the possible presence of carbonate impurities. On the other hand, the Fe_2_O_3_ content (0.0–2.7%) suggests the presence of oxides, which are likely present in an accessory manner.

The chemical composition of the analyzed carbonate aggregates was predominantly constituted by CaO (28.3–56.0%) and CO_2_ (34.7–45.9%), which constitute calcite (CaCO_3_) or dolomite (CaMg(CO_3_)_2_), given that MgO is present in proportions of up to 20% in this group. The presence of SiO_2_ (0.0–19.4%) and, to a lesser extent, Al_2_O_3_ (0.0–3.8%) suggests the presence of possible impurities in these samples.

On the other hand, the volcanic aggregates showed a SiO_2_ percentage range of 42.0–61.1%, with other elements present, such as Al_2_O_3_ (12.5–17.3%), Fe_2_O_3_ (4.5–14.3%), CaO (3.8–11.6%), MgO (2.3–10.6%), Na_2_O (1.9–5.4%), K_2_O (1.5–2.4%), and TiO_2_ (0.7–4.0%).

Finally, the set of granite aggregates exhibited a different composition range. SiO_2_ (59.8–74.6%) was the most abundant element, with the other oxides present being Al_2_O_3_ (12.6–16.2%), CaO (0.6–3.9%), K_2_O (3.6–6.2%), and Na_2_O (2.7–5.0%). Fe_2_O_3_ (1.2–4.7%), MgO (0.0–3.0%), and TiO_2_ (0.2–0.8%) were also present. All these results are in agreement with previous studies [6,9,46,47,48].

The ranges of the activity concentrations of the natural radionuclides evidenced the influence of the geological origin of the aggregates. The radionuclides from the uranium and thorium series were found in higher concentrations in granite-origin aggregates, followed by volcanic, carbonate, and silica aggregates. Conversely, ^40^K was present in higher concentrations in the set of granite aggregates (785–1272 Bq kg^−1^), followed by the set of silica aggregates (2–676 Bq kg^−1^), volcanic aggregates (79–548 Bq kg^−1^), and finally those of a carbonate composition (3–153 Bq kg^−1^). The obtained ranges showed less dispersion in granite aggregates compared to the other aggregates.

### 3.2. Mineralogy of Aggregates

**Figure 5:**
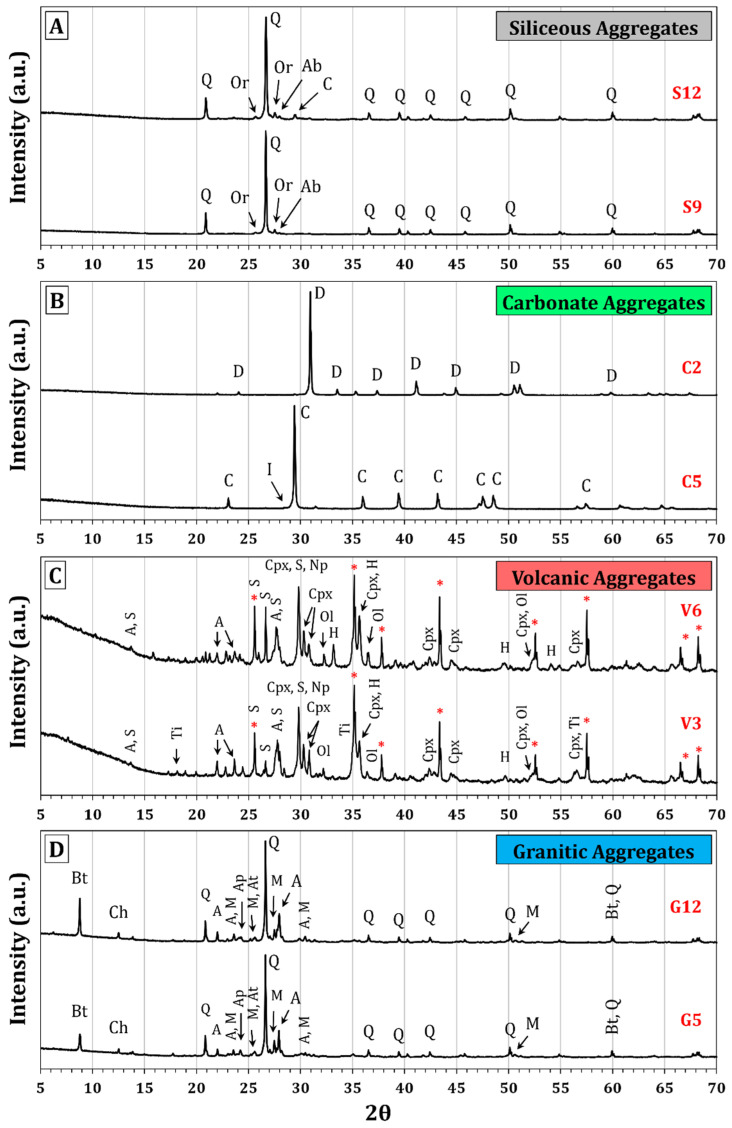
Diffractograms of aggregate samples: (**A**) silica, (**B**) carbonate, (**C**) volcanic, and (**D**) granite. The identified mineral phases were Q = Quartz (SiO_2_) (COD 1011097), Or = Orthoclase (K Feldspar) (KAlSi_3_O_8_) (COD 9000161), Ab = Albite (Plagioclase) (NaAlSi_3_O_8_) (COD 9000681), C = Calcite (CaCO_3_) (COD 1010962), D = Dolomite (CaMg(CO_3_)_2_) (COD 1200014), I = Illite ((K,H_3_O)(Al,Mg,Fe)_2_(Si,Al)_4_O_10_[(OH)_2_,(H_2_O)]) (COD 9013718), A = Anorthite (Plagioclase) (CaAl_2_Si_2_O_8_) (COD 9000747), S = Sanidine (K Feldspar) ((K,Na)(Si,Al)_4_O_8_) (COD 9001103), Cpx = Clinopyroxene, Diopside (CaMgSi_2_O_6_) (COD 9001307), Np = Nepheline (NaAlSiO_4_) (COD 9004995), Ol = Olivine (Mg_2_SiO_4_) (COD 9000166), H = Hematite (Fe_2_O_3_) (COD 9000139), Ti = Titanomagnetite (Fe_3−x_TiO_4_) (COD 9000933), Ch = Chlorite, Chamosite ((Fe,Mg)_5_Al(Si_3_Al)O_10_(OH)_8_) (COD 9009233), Bt = Biotite ((K(Mg,Fe)_3_(AlSi_3_O_10_)(OH)_2_) (COD 9000468), M = Microcline (K Feldspar) (KAlSi_3_O_8_) (COD 9000189), Ap = Apatite (Ca_5_(PO_4_)_3_(F, Cl, OH)) (COD 9010383), At = Anatase (TiO_2_) (COD 1010942). The peaks associated with the internal pattern of corundum (Al_2_O_3_) are marked as (*).

Figure 5 shows the diffractograms of the selected samples from each type of aggregate included in this study, along with the identification of their corresponding mineral phases. The peaks associated with the internal pattern of corundum (Al_2_O_3_) are marked as (*). Table 2 presents the results of the quantification of the mineral phases present in the analyzed samples, obtained via Rietveld refinement.

The silica aggregates are predominantly composed of quartz, given the high percentage of SiO_2_ present in this set of samples. Additionally, variable amounts of potassium feldspars such as orthoclase and sodium feldspars such as albite were found (13.77% and 9.71% in S9 and S12, respectively), which is consistent with the presence of variable amounts of Al_2_O_3_, K_2_O, and Na_2_O. In sample S12, calcite was identified at 4.24%, which explains the elevated loss on ignition (LoI) (up to 5%) present in some samples of this set (see Table S1).

The carbonate aggregates predominantly presented calcite (C5) or dolomite (C2), with the minor presence of quartz in C2 and illite in C5.

On the other hand, the volcanic aggregates exhibited variable content of Al_2_O_3_, Fe_2_O_3_, CaO, MgO, Na_2_O, and K_2_O, which, in combination with SiO_2_, form a wide variety of silicate mineral phases, such as feldspars (plagioclase, sanidine), feldspathoids (nepheline), pyroxenes, and olivine. Additionally, it is common to find other accessory minerals, such as hematite and minerals rich in TiO_2_ (rutile, ilmenite, titanomagnetite, etc.).

Finally, the granite aggregates had a heterogeneous mineralogical composition, predominantly rich in quartz due to the high SiO_2_ content. Moreover, the SiO_2_ in combination with the Al_2_O_3_, CaO, Na_2_O, and K_2_O present in these rocks justifies the presence of potassium feldspars and plagioclases. The content of Fe_2_O_3_ and MgO also accounts for the presence of various phyllosilicates, such as biotite and chlorite. Lastly, accessory minerals such as anatase and apatite were found, associated with the variable TiO_2_ and P_2_O_5_ content, as well as zircon; although the diffractograms do not show intense peaks, this justifies its presence in trace amounts due to the content of ZrO_2_ (Table S1).

The mineralogical composition found in these samples is consistent with the scientific literature [49,50] and aligns with the results obtained through XRF (Table S1).

### 3.3. Correlation Between Chemical Composition and Activity Concentration

**Figure 6:**
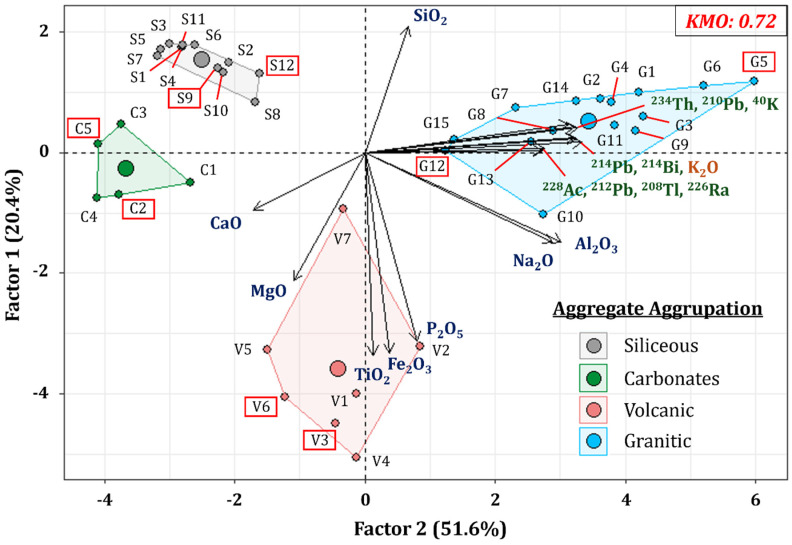
HJ-Biplot of the principal component analysis (PCA) relating the variables of the chemical composition and activity concentrations of the radionuclides from the natural uranium and thorium radioactive series, as well as ^40^K, for the aggregate set included in the study. This analysis achieved a KMO suitability index of 0.72. Samples where XRD was performed and the Rietveld method was applied are highlighted in red.

The results regarding the chemical composition and activity concentrations were analyzed using principal component analysis (PCA) and represented in an HJ-Biplot graph (Figure 6). The figure illustrates the two factors identified for the four types of aggregates and the clusters derived from the sample scores. The samples selected for the subsequent validation of the method are highlighted with a red box.

The variables excluded from the PCA were MnO, SO_3_, CO_2_, and ZrO_2_, achieving a KMO suitability index of 0.72, which indicates a satisfactory degree of correlation [51]. The two main factors identified in the PCA explain 72% of the variance. Factor 1 accounts for 20.4% of the total variance and is associated with the chemical composition variables SiO_2_, Fe_2_O_3_, TiO_2_, and P_2_O_5_. In contrast, Factor 2 explains 51.6% of the variance and is directly related to the activity concentration variables of the radionuclides included in the study (^234^Th, ^226^Ra, ^214^Pb, ^214^Bi, ^210^Pb, ^228^Ac, ^212^Pb, ^208^Tl, and ^40^K), as well as the content of K_2_O.

The obtained HJ-Biplot, which is shown in Figure 6, reveals a strong correlation between the activity concentration variables of radionuclides from the uranium series (^234^Th, ^226^Ra, ^214^Pb, ^214^Bi, ^210^Pb) and the thorium series (^228^Ac, ^212^Pb, ^208^Tl), as well as ^40^K and K_2_O content. This set of variables is less correlated with Al_2_O_3_ and Na_2_O, whose proximity to the K_2_O vector could be explained by the involvement of these elements in the formation of alkaline feldspars. However, an inverse correlation was observed in the case of CaO, indicating that, as the percentage of CaO increases, the activity concentrations of the determined radionuclides decrease. On the other hand, the vectors corresponding to the chemical variables Fe_2_O_3_, TiO_2_, and P_2_O_5_ formed angles of 90° with those variables related to the radionuclide content, indicating the absence of a correlation between them. The samples represented in the HJ-Biplot were grouped coherently according to their chemical compositions and nature, constituting the four groupings observed in Figure 6, i.e., siliceous, carbonate, volcanic, and granitic aggregates, which were positioned away from the coordinate center, thus making the groupings statistically representative.

The cluster of siliceous aggregates (quartz–feldspathic) exhibited low dispersion and presented an inverse correlation with Al_2_O_3_, Na_2_O, K_2_O, and ^40^K, as well as with the radionuclides from the uranium and thorium series. The carbonate aggregates were located close to the siliceous ones, suggesting that they would display similar behavior regarding the radionuclide content. However, this grouping showed a greater affinity for CaO, a fundamental element in this set of samples.

The grouping obtained from volcanic aggregates demonstrated greater dispersion in the sample scores, reflecting an affinity for the percentages of MgO, TiO_2_, Fe_2_O_3_, and P_2_O_5_. The projection of this group of samples in the HJ-Biplot forms an angle of approximately 90° with respect to the vectors representing the radionuclides from the uranium and thorium radioactive series, as well as ^40^K. Therefore, the radionuclide content of this group would show a low or no correlation with the chemical composition.

Finally, the grouping formed by granitic aggregates showed significant weight in Factor 1, indicating a strong correlation with the radionuclides from the uranium and thorium series, as well as with the content of K_2_O and ^40^K. The correlation found between granites and the radionuclide content is consistent with other studies that have associated the high radioactivity of granitic rocks with the presence of potassium-rich minerals (orthoclase) and accessory minerals rich in radionuclides (zircon, apatite) [52].

### 3.4. Predictive Models of Activity Concentrations of ^226^Ra, ^232^Th (^212^Pb), and ^40^K Based on Chemical Composition Parameters

**Figure 7:**
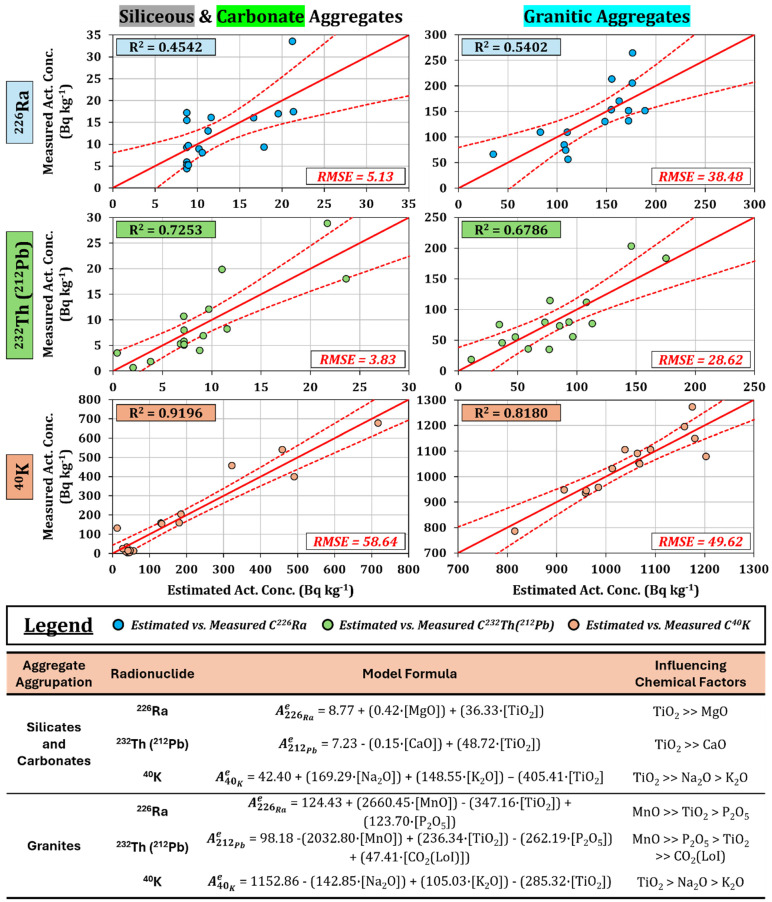
Predictive models of the activity concentrations of the radionuclides ^226^Ra, ^232^Th (^212^Pb), and ^40^K in granitic and siliceous/carbonate aggregates based on their chemical compositions. The red dashed lines indicate the upper and lower limits of the confidence band for each generated model. The RMSE index reflects the accuracy by indicating the average error. At the bottom, the expressions of the proposed models are shown, along with the chemical variables ordered from strongest to weakest influence in the models.

Figure 7 shows the linear fits representing the activity concentrations obtained through gamma spectrometry as a function of the predicted values from the different models. Additionally, each fit includes the confidence intervals of the linear adjustments, which highlight the results that overlap with the linear fit. The predicted values fall significantly within these confidence intervals, indicating that the information provided by the fits aligns with the experimental measurements conducted. The figure also includes the values of the root mean square error (RMSE), which are used to evaluate the dispersion of the obtained models. On the other hand, the figure illustrates the models derived through multiple regression analysis, which estimate the activity concentrations of the radionuclides necessary to calculate the activity concentration indices (ACIs) of siliceous, carbonate, and granitic aggregates based on their chemical compositions. Additionally, Figure 7 shows the expressions of the proposed models, along with their chemical variables ordered by their influence from highest to lowest. Conversely, predictive models were not developed for the volcanic aggregate set due to the lack of correlation between the activity concentrations of radionuclides and the chemical composition (Figure 6).

Siliceous and carbonate aggregates were analyzed as a single dataset due to the similarity identified in the principal component analysis (PCA), while the second dataset analyzed consisted of granitic aggregates.

Table 3 shows the results obtained from the Student’s *t*-tests and Fisher’s F-tests, which were used to verify whether the means and variances were statistically significant between the experimental values and those estimated by the models. The F- and *t*-values exceeded the chosen significance level (α = 0.05), indicating that there were no significant differences between the means and variances of both sets of results.

The proposed model for ^226^Ra from the siliceous and carbonate aggregate set achieved a coefficient of determination (R^2^) of 0.4542, with an RMSE of 5.13, indicating dispersion of 41.30% based on an average value of ^226^Ra of 12.42 Bq kg^−1^ for these samples. On the other hand, the model obtained for ^232^Th (^212^Pb) yielded a coefficient of determination of 0.725 and an RMSE of 3.83, indicating dispersion of 45.29% based on an average value for ^212^Pb of 8.45 Bq kg^−1^. The chemical variable with the greatest weight in the models for ^226^Ra and ^232^Th was the percentage of TiO_2_, which is related to the presence of accessory minerals such as ilmenite and rutile. This mineralogy, along with other heavy minerals, is associated with the presence of natural radionuclides from the natural radioactive series of uranium and thorium [53,54,55]. Furthermore, due to their high resistance to abrasion, these minerals tend to be part of the accessory mineralogy in soils, sediments, and detrital rocks through accumulation [54,56].

The predictive model for the activity concentration of ^40^K determined for the siliceous and carbonate aggregate set achieved an R^2^ of 0.920, showing the correctness of the trend in the experimental points relative to the obtained model. The RMSE value obtained in this model was 58.64, corresponding to 33.52% dispersion regarding the average experimental value of ^40^K of 174.98 Bq kg^−1^. The most influential chemical factors in this model were ranked from highest to lowest weight, with the percentages of TiO_2_ (even at low concentrations), Na_2_O, and K_2_O. Na_2_O and K_2_O are fundamental compounds in feldspars, so the positive contribution to the model would be related to the mineralogical proportion of their solid solution. However, the content of TiO_2_ penalized the estimation of ^40^K in the model. In other words, the presence of feldspar impurities, micas, oxides, and other accessory minerals would be related to a higher activity concentration of radionuclides from the ^238^U and ^234^Th.

The model determined for ^226^Ra in granite aggregates achieved a coefficient of determination (R^2^) and RMSE of 0.5402 and 38.48, respectively, corresponding to dispersion of 27.94% relative to the average experimental value of ^226^Ra, which is 137.72 Bq kg^−1^. On the other hand, the models determined for ^232^Th (^212^Pb) yielded R^2^ and RMSE values of 0.6786 and 28.62, corresponding to dispersion of 34.71% relative to the average experimental value of ^232^Th (^212^Pb), which is 82.46 Bq kg^−1^. Both models showed that the factor with the greatest weight was the percentage of MnO, followed by TiO_2_ and P_2_O_5_. These results reflect that the estimation of both ^226^Ra and ^212^Pb does not depend on the percentage of major elements but rather on those that are part of the accessory mineralogy of the granites.

The accessory minerals that imply the presence of natural radionuclides in granitic igneous rocks are those whose composition includes the phosphate group (monazite and apatite), titanium oxides (rutile, ilmenite, and titanite), and iron oxides (hematite) [57]. Additionally, pyrolusite (MnO_2_) is indirectly related to the content of radionuclides from the uranium series due to its high oxidative capacity, oxidizing U^4+^ to UO_2_^2+^ in alteration environments and absorbing them on its surface [58]. For this reason, pyrolusite is sometimes associated with uranium deposits in granitic regions [59]. However, the presence of this mineral could not be confirmed by X-ray diffraction (XRD) due to the weak intensity of its diffraction peaks, which may have been masked by the presence of other more abundant minerals (quartz and feldspars). Nevertheless, the MnO content found through X-ray fluorescence (FRX) in these aggregates (Table S1) justifies the accessory presence of Mn oxides, which positively influence the estimation of ^226^Ra (uranium series) and negatively affect the predictive model of ^232^Th (^212^Pb).

Finally, the estimation model for ^40^K achieved an R^2^ of 0.818 and an RMSE of 49.62, corresponding to dispersion of 4.74%, determined from the average activity concentration value of 1046.49 Bq kg^−1^ for the experimental ^40^K, confirming the low dispersion of the results estimated by this model. The chemical compounds that influenced this model, in order of weight, were TiO_2_, Na_2_O, and K_2_O, with TiO_2_ showing a negative influence on the estimation, while K_2_O had a positive impact. This inverse relationship is consistent with the processes of magmatic differentiation in granites, where, as minerals rich in TiO_2_ (ilmenite, rutile) precipitate, the magma evolves and becomes enriched in K_2_O, which ultimately forms feldspars [60].

### 3.5. Validation of the Achieved Models

**Figure 8:**
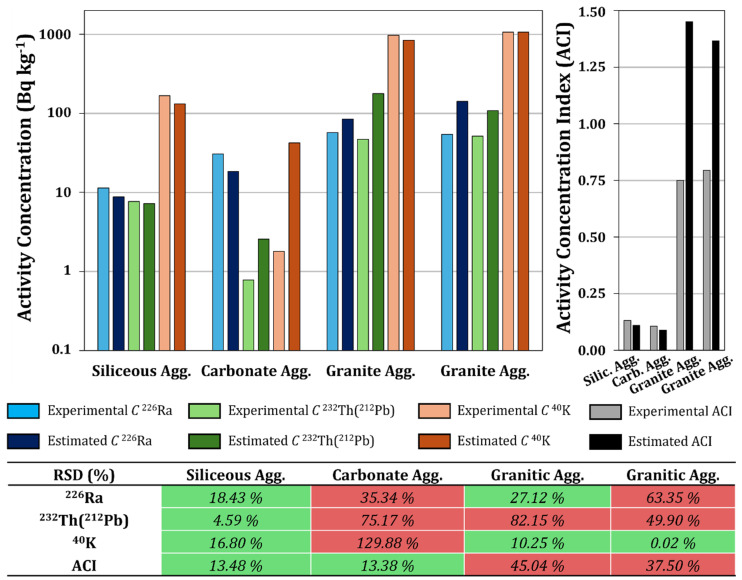
Comparison between the estimated activity concentration results and those obtained through gamma spectrometry for the radionuclides ^226^Ra, ^232^Th (^212^Pb), and ^40^K from siliceous, carbonate, and granitic aggregates. Additionally, the ACI calculated using the estimated values was compared with that calculated from experimental data. The RSD (%) values that met the acceptance criterion assigned for the models (RSD (%) ≤ 30%) are shaded in green, while those that exceeded the threshold value are shaded in red.

The validation of the developed models was performed by analyzing aggregate samples that were not included in the statistical analysis, following the methodology described in Section 2.2.4. These samples included one sample of siliceous origin, one sample of carbonate origin, and two samples of granitic origin. Figure 8 shows the activity concentrations of ^226^Ra, ^232^Th (^212^Pb), and ^40^K obtained for the siliceous, carbonaceous, and granitic aggregates, as well as the values of the activity concentration index (ACI) derived from these values. Additionally, the figure contains the relative standard deviation (RSD%) values obtained for each of the estimated activity concentrations and ACIs, which indicate the relative percentage differences between the experimental values and those obtained from the proposed models.

Fifty percent of the RSD (%) values were below 30%, meeting the acceptance criterion established for the models. The RSD (%) obtained for the models determined for the siliceous aggregate set was satisfactory for both the ACI and the activity concentrations of ^226^Ra, ^212^Pb, and ^40^K in all validation samples. On the other hand, the models determined for the carbonate aggregate set were not satisfactory, as all estimated activity concentration values for ^226^Ra, ^212^Pb, and ^40^K were overestimated by the models. The discordant results may be attributed to the low proportions of TiO_2_, Na_2_O, and K_2_O in this type of rock, whose presence is associated with the content of natural radionuclides, leading to the low predictive capacity of the model for this type of aggregate. However, the RSD (%) criterion for the calculation of the ACI in this set was met. Therefore, although the models do not accurately estimate the activity concentrations of ^226^Ra, ^212^Pb, and ^40^K, they do so for the ACI, which could provide valuable information for the selection of critical samples.

Regarding the granitic aggregate set, the RSD (%) values obtained for ^40^K achieved a 100% satisfactory rate. However, the models related to the activity concentration of ^226^Ra and especially to ^232^Th (^212^Pb) did not meet the acceptance criterion, with satisfactory rates of 50% and 0%, respectively. In all cases, the estimated values were higher than the experimental values. This overestimation may have been due to the relationship between the activity concentration and the percentage of minor elements such as MnO, P_2_O_5_, and TiO_2_. On the other hand, the models achieved a 50% satisfactory rate for the RSD (%) concerning the ACI, allowing for the accurate estimation of the set of radionuclides considered in this index.

Finally, the ACI values obtained through the proposed models for siliceous and carbonate aggregates were lower than those determined from the experimental activity concentrations. Nevertheless, they provide a reasonably accurate estimate to assess their suitability concerning the current legislation. In the case of granitic aggregates, the ACIs obtained through the proposed models were higher than those obtained from the experimental activity concentrations, with the differences reaching up to 45%. This significant difference is attributed to the high heterogeneity in the chemical and mineralogical compositions of granites, which complicates the estimation of the activity concentrations of ^226^Ra, ^232^Th, and ^40^K. Furthermore, as previously indicated, the activity concentrations of these radionuclides are fundamentally associated with the accessory mineralogy of the granites and, therefore, are not related to the chemical composition regarding the major elements in this case.

## 4. Conclusions

This study examined the relationship between the chemical composition and the activity concentrations of natural radionuclides present in four types of aggregates commonly used in mortars and concretes: eight of the siliceous type, five of the carbonate type, seven of volcanic origin, and fifteen of the granitic type. In addition, the different mineralogical phases obtained by X-ray diffraction (XRD) have been correlated with the chemical composition and radioactive content to explain and understand the various relationships observed.

The chemical composition obtained through X-ray fluorescence (XRF) revealed high values of SiO_2_ for the siliceous aggregates, reaching up to 99.2%. Additionally, the Al_2_O_3_ values ranged between 0.2% and 12.1% due to the presence of feldspars. The carbonatic aggregates exhibited high content of CaO, which reached up to 55.4%, and a CO_2_ percentage of 43.5%; this is associated with carbonates, linked to the presence of calcite and dolomite. The chemical composition of the volcanic aggregates was more complex, with the main characteristic being a range of SiO_2_ between 42.0% and 61.1%, along with Al_2_O_3_ at 15.8% and Fe_2_O_3_ at 10.2%. The composition of the granitic aggregates was more homogeneous than that of the other aggregates, with a SiO_2_ range between 59.8% and 74.6% and percentages of Al_2_O_3_ and Na_2_O reaching up to 15.0% and 5.9%, respectively. Regarding the mineral phases obtained through X-ray diffraction (XRD), it was observed that, in the siliceous aggregates, there was a predominant quartz phase with a SiO_2_ range varying between 78.9% and 99.2%, as well as the presence of potassium and sodium feldspars. The carbonatic aggregates contained calcite and dolomite as the main phases, along with a small percentage of quartz and illite. The volcanic aggregates displayed greater variability in the determined mineral phases, with a SiO_2_ percentage ranging from 42.0% to 61.1%, along with smaller proportions of feldspars, pyroxenes, and olivine. The granitic aggregates showed the presence of mineral phases that indicated a greater contribution of natural radionuclides, such as zircon and monazite. The content of natural radionuclides was as expected, with the aggregates containing the highest levels of natural radionuclides being the granitic aggregates in both the uranium and thorium series. However, in the remaining aggregates, the concentrations of these radionuclides were lower, with only significant concentrations of ^40^K found, ranging from 2 to 676 Bq kg^−1^ for the siliceous aggregates, from 79 to 548 Bq kg^−1^ for the volcanic aggregates, and between 3 and 153 Bq kg^−1^ for the carbonatic aggregates.

The correlations between the chemical compositions of the aggregates and the activity concentrations were determined using an HJ-Biplot graph, which compiled the results from the principal component analysis (PCA). The graph accurately grouped the aggregates into the four investigated categories. The variables excluded from the analysis were MnO, SO_3_, CO_2_, and ZrO_2_, resulting in a KMO adequacy index of 0.72, confirming the satisfactory correlation. The two factors obtained explained 72% of the variance, with Factor 1 associated with SiO_2_, Fe_2_O_3_, TiO_2_, and P_2_O_5_, while Factor 2 was related to the natural radionuclides and K_2_O. Additionally, a strong correlation was observed between the concentrations of radionuclides from the uranium and thorium series, as well as with ^40^K and K_2_O. On the other hand, an inverse correlation was found between CaO and the natural radionuclides present.

The multiple regression analysis enabled the development of predictive models to estimate the activity concentrations of radionuclides in siliceous, carbonatic, and granitic aggregates based on their chemical compositions. The models developed for siliceous and carbonatic aggregates demonstrated a significant correlation with natural radionuclides. However, a weak correlation was observed for volcanic aggregates, preventing the development of a model. The models for siliceous and carbonatic aggregates achieved satisfactory coefficients of determination (R^2^) of 0.920 and 0.454 for ^226^Ra in siliceous and carbonatic aggregates, respectively. Conversely, the models for granitic aggregates exhibited overestimations in the activity concentrations of natural radionuclides due to the complex relationship between the radionuclides and the accessory mineralogy, yielding R^2^ values of 0.5402 for ^226^Ra and 0.6786 for ^212^Pb. Model validation revealed that, while some models met the acceptance criteria (50% of the results), others failed to accurately estimate the experimental activity concentrations, with this issue being more pronounced for carbonatic and granitic aggregates. Despite these limitations, the calculated activity concentration indices (ACIs) provide valuable estimates for the evaluation of the suitability of these aggregates in accordance with the current regulations.

The future of forthcoming research aimed at expanding this investigation must necessarily involve correlating the activity concentrations of natural radionuclides with the chemical composition regarding minor and trace elements, obtained through methods with greater analytical sensitivity, as well as with the mineralogical compositions of the aggregates.

## Figures and Tables

**Table 1:** Energy (keV) and emission probabilities (%) of the radionuclides analyzed in this study.

Radioactive Series	Radionuclide	Energy (keV)	Photons/100 Disintegrations
Uranium Series	^234^Th	63.30 (2)	3.75 (8)
	^226^Ra	186.211 (13)	3.555 (19)
	^214^Pb	351.932 (2)	35.60 (7)
	^214^Bi	609.312 (7)	45.49 (19)
		1120.287 (10)	14.91 (3)
		1764.494 (14)	15.31 (5)
	^210^Pb	46.539 (1)	4.252 (40)
Thorium Series	^228^Ac	911.196 (6)	26.2 (8)
	^212^Pb	238.632 (2)	43.6 (5)
	^208^Tl	583.187 (2)	85.0 (5)
Potassium	^40^K	1460.822 (6)	10.55 (11)

**Table 2:** Weight (wt.%) of mineral phases found in the analyzed aggregate samples. In the volcanic aggregates, the % of amorphous volume has been included. The R_wp_ index reflects the quality of the fit between the experimental data and the theoretical model obtained via Rietveld refinement.

Mineral Classification	Phases (%)	Siliceous Agg.		Carbonate Agg.		Volcanic Agg.		Granitic Agg.	
		S9	S12	C2	C5	V3	V6	G5	G12
Silicates	Quartz	83.77	70.35	0.15				30.58	26.05
	K Feldspar	2.46	15.70			1.14	6.52	30.72	31.69
	Plagioclase	13.77	9.71			16.10	13.74	22.68	27.55
	Biotite *							13.36	11.53
	Chlorite							0.32	0.42
	Illite *				0.37				
	Diopside					37.88	37.33		
	Nepheline					0.92	0.74		
	Olivine					1.43	2.67		
	Zircon							0.14	0.20
Carbonates	Calcite		4.24	0.28	99.63				
	Dolomite			99.57					
Oxides	Hematite					2.83	4.18		
	Titanomagnetite					3.29			
	Anatase							0.80	0.78
Phosphates	Apatite							1.40	1.78
Amorphous						36.40	34.81		
R_wp_		13.90	9.99	11.56	11.35	3.92	4.26	10.65	10.68

* Minerals of the mica group.

**Table 3:** *p*-Values from the paired Student’s *t*-test and Fisher’s F-test, which evaluated the accuracy and precision of the estimated activity concentration results compared to those obtained through gamma spectrometry. Both tests were performed at a significance level of α = 0.05.

	Radionuclide	Student’s *t*-Test	Fisher’s F-Test
Siliceous and Carbonate Aggregates	^226^Ra	1.000	0.125
	^232^Th(^212^Pb)	1.000	0.084
	^40^K	1.000	0.528
Granite Aggregates	^226^Ra	1.000	0.261
	^232^Th(^212^Pb)	1.000	0.477
	^40^K	0.999	0.712

## Data Availability

The original contributions presented in this study are included in the article/Supplementary Material. Further inquiries can be directed to the corresponding author.

## Supplementary Materials

The following supporting information can be downloaded at: https://www.mdpi.com/article/10.3390/ma18061369/s1, Table S1. Chemical compositions (wt.%) of the analyzed aggregates obtained by XRF; Table S2. Activity concentrations (Bq kg^−1^) of the radionuclides from the natural radioactive series of uranium and thorium and ^40^K from the analyzed aggregate samples; Table S3. Chemical compositions (wt.%) of the aggregate samples used in the validation of the models; Table S4. Activity concentrations (Bq kg^−1^) of the aggregate samples used in the validation of the models.
