# Supplementary material for: Prediction Models for Radiological Characterization of Natural Aggregates Based on Chemical Composition and Mineralogy
Source: Materials (Basel). 2025 Mar 20;18(6):1369. doi: 10.3390/ma18061369 (PMC11944134; doi:10.3390/ma18061369)
Supplement: Supplementary file 1 [file materials-18-01369-s001.zip › materials-3511397-supplementary.pdf]

SUPPLEMENTARY MATERIAL

Table S1. *Chemical composition (wt.%) of the aggregates obtained by XRF.*

Sample	SiO_2	CaO	Al_2O_3	Fe_2O_3	MgO	MnO	Na_2O	K_2O	TiO_2	P_2O_5	SO_3	ZrO_2	LoI*
**S1**	96.80	0.10	1.50	0.40	0.00	0.00	0.00	0.60	0.00	0.00	0.00	0.00	0.60
**S2**	91.93	0.34	4.52	0.17	0.00	0.00	0.00	1.89	0.00	0.12	0.00	0.00	1.05
**S3**	99.16	0.06	0.86	0.01	0.00	0.00	0.00	0.10	0.04	0.00	0.00	0.00	0.13
**S4**	95.81	0.13	1.93	0.04	0.01	0.00	0.08	0.84	0.00	0.00	0.00	0.00	0.21
**S5**	94.69	2.49	0.76	0.31	0.00	0.00	0.00	0.10	0.00	0.00	0.00	0.00	1.65
**S6**	98.02	0.05	1.05	0.14	0.00	0.00	0.00	0.19	0.08	0.00	0.00	0.01	0.45
**S7**	87.95	5.67	0.99	0.12	0.00	0.00	0.00	0.10	0.05	0.00	0.00	0.01	5.11
**S8**	83.26	0.98	7.08	2.67	0.58	0.05	1.74	1.97	0.34	0.00	0.00	0.01	1.31
**S9**	92.26	0.19	4.04	0.24	0.00	0.57	2.46	0.00	0.00	0.00	0.00	0.00	0.23
**S10**	92.26	0.00	3.51	2.36	0.00	0.00	0.00	0.62	0.30	0.00	0.00	0.05	0.91
**S11**	97.01	0.06	1.64	0.19	0.00	0.00	0.00	0.96	0.00	0.00	0.00	0.00	0.13
**S12**	78.89	5.77	6.02	0.56	0.00	0.00	0.98	3.62	0.07	0.00	0.00	0.00	4.09
**C1**	14.69	28.30	3.82	1.26	14.34	0.02	0.38	0.66	0.18	0.00	0.79	0.03	35.49
**C2**	0.99	34.11	0.76	0.07	18.68	0.00	0.00	0.03	0.00	0.00	0.03	0.00	45.30
**C3**	19.40	44.88	0.00	0.34	0.45	0.00	0.00	0.04	0.00	0.00	0.14	0.00	34.73
**C4**	1.23	33.99	0.00	0.26	18.52	0.00	0.00	0.00	0.04	0.00	0.00	0.00	45.93
**C5**	0.00	56.03	0.23	0.02	0.40	0.00	0.00	0.00	0.00	0.00	0.00	0.00	43.31
**V1**	45.14	8.60	13.20	10.87	7.33	0.22	4.33	2.33	2.94	0.84	0.00	0.00	3.65
**V2**	45.86	9.86	13.10	9.41	5.80	0.18	4.26	2.43	2.55	0.72	0.00	0.00	5.20
**V3**	41.98	11.59	13.52	13.72	8.73	0.18	4.30	1.46	3.53	0.69	0.00	0.03	0.00
**V4**	42.60	11.40	14.20	13.80	7.00	0.21	4.07	1.64	3.99	0.99	0.00	0.00	0.00
**V5**	44.57	9.23	12.46	12.15	10.63	0.14	1.93	1.60	2.51	0.44	0.00	0.04	4.06
**V6**	44.34	10.88	12.87	14.28	6.83	0.18	2.80	1.81	3.36	0.60	0.06	0.07	1.50
**V7**	61.09	3.75	17.33	4.47	2.28	0.06	5.35	2.29	0.70	0.20	0.00	0.02	2.40
**G1**	73.51	0.86	14.12	1.65	0.50	0.04	2.86	4.89	0.27	0.15	0.00	0.00	1.10
**G2**	74.60	1.30	12.77	2.50	0.39	0.05	3.15	4.31	0.32	0.07	0.00	0.00	0.50
**G3**	69.72	2.84	12.77	3.08	2.84	0.06	2.71	4.70	0.35	0.08	0.00	0.00	2.90
**G4**	69.95	1.02	15.50	2.01	0.43	0.04	3.86	6.17	0.26	0.00	0.00	0.01	0.76
**G5**	73.94	0.71	14.36	1.41	0.00	0.03	3.82	4.80	0.16	0.24	0.00	0.01	0.49

**G6**	71.23	1.51	13.32	1.94	0.40	0.05	4.97	5.82	0.24	0.00	0.00	0.02	0.46
**G7**	73.66	1.34	12.58	2.53	0.38	0.04	3.95	4.93	0.33	0.00	0.00	0.03	0.24
**G8**	72.26	0.56	14.54	1.40	0.00	0.03	4.48	5.47	0.17	0.39	0.00	0.00	0.69
**G9**	71.46	1.30	14.85	1.83	0.61	0.00	4.18	4.72	0.33	0.20	0.00	0.02	0.47
**G10**	59.85	3.94	16.21	4.65	2.95	0.07	4.17	6.02	0.81	0.53	0.00	0.04	0.66
**G11**	70.64	0.98	14.47	1.90	0.36	0.04	4.56	6.05	0.24	0.26	0.00	0.02	0.50
**G12**	70.44	2.06	15.09	2.40	0.68	0.04	4.24	3.63	0.40	0.22	0.00	0.02	0.77
**G13**	69.24	2.09	14.62	3.25	0.93	0.06	4.01	4.64	0.53	0.16	0.00	0.02	0.39
**G14**	72.30	0.79	14.47	2.03	0.35	0.04	3.45	5.76	0.22	0.12	0.00	0.01	0.36
**G15**	73.71	0.57	14.70	1.15	0.00	0.00	3.61	4.83	0.19	0.40	0.00	0.01	0.82

**LoI: Lost on Ignition. S= Siliceous, C= Carbonates, V= Volcanic, G= Granitic.*

Table S2. *Activity concentration (Bq kg^{-1}) of the radionuclides of the natural radioactive series of uranium and thorium, and ^{40}K of the analysed aggregate samples..*

**Series**	**^{238}U Radioactive Serie**					**^{232}Th Radioactive Serie**			**^{40}K**
**Sample**	**^{234}Th**	**^{226}Ra**	**^{214}Pb**	**^{214}Bi**	**^{210}Pb**	**^{228}Ac**	**^{212}Pb**	**^{208}Tl**	
**S1**	-	15.4 ± 5.8	4.68 ± 0.61	-	7.2 ± 1.5	7.01 ± 0.49	7.9 ± 1.0	2.89 ± 0.20	157 ± 16
**S2**	13.85 ± 3.88	17.15 ± 6.42	10.6 ± 1.3	9.25 ± 0.79	15.45 ± 4.58	9.63 ± 0.88	10.65 ± 1.24	4.33 ± 0.55	456 ± 29
**S3**	6.75 ± 2.54	8.9 ± 4.0	6.3 ± 0.8	6.1 ± 0.7	10 ± 3	5.83 ± 0.45	6.85 ± 0.81	2.55 ± 0.29	< 2.7
**S4**	5.9 ± 3.9	9.2 ± 4.6	3.12 ± 0.88	3.01 ± 0.50	6.1 ± 2.9	4.31 ± 0.50	5.01 ± 0.66	2.23 ± 0.27	157.5 ± 10.5
**S5**	5.01 ± 1.16	5.85 ± 2.22	2.06 ± 1.08	1.93 ± 1.10	4.4 ± 1.3	4.53 ± 0.21	5.25 ± 0.29	2.06 ± 1.01	11.14 ± 2.19
**S6**	15.71 ± 1.69	16.05 ± 2.28	14.71 ± 0.77	13.56 ± 0.96	16.36 ± 1.81	18.68 ± 1.10	19.79 ± 1.33	7.09 ± 2.82	31.11 ± 4.74
**S7**	5.4 ± 1.1	8.0 ± 1.9	2.52 ± 0.52	2.43 ± 0.48	4.60 ± 0.86	3.25 ± 0.45	3.98 ± 0.45	1.41 ± 0.11	8.0 ± 2.0
**S8**	14.5 ± 4.7	17.4 ± 6.9	11.65 ± 0.85	10.08 ± 0.56	15.75 ± 5.48	16.55 ± 1.05	17.95 ± 1.05	6.61 ± 0.45	398.5 ± 24.2
**S9**	< 10.5	< 8.4	3.64 ± 0.36	3.69 ± 0.35	< 8.5	4.25 ± 0.48	5.74 ± 0.40	1.75 ± 0.33	539 ± 33
**S10**	27.3 ± 4.5	16.95 ± 5.60	19.3 ± 1.2	18.15 ± 0.92	20 ± 5	23.55 ± 1.31	28.8 ± 1.7	9.17 ± 0.62	130 ± 8
**S11**	7.6 ± 7.5	< 8.2	3.42 ± 0.32	3.21 ± 0.29	5.2 ± 3.6	3.61 ± 0.35	5.14 ± 0.35	1.72 ± 0.16	203.5 ± 12.7
**S12**	17.3 ± 3.6	13 ± 11	10.8 ± 1.0	9.64 ± 1.04	14.8 ± 7.8	9.65 ± 0.86	12.05 ± 0.95	4.25 ± 0.44	676 ± 43
**C1**	47.85 ± 6.92	33.5 ± 10.1	40.3 ± 2.6	38.5 ± 2.9	59.5 ± 7.1	10.95 ± 1.05	8.15 ± 4.85	4.83 ± 0.46	152.5 ± 10.6
**C2**	35 ± 7	16 ± 8	22.95 ± 1.41	21.3 ± 1.0	29.9 ± 7.2	< 1.9	< 0.9	< 0.6	< 5.0

**C3**	13.85 ± 4.06	9.6 ± 4.4	10.02 ± 0.69	9.35 ± 0.54	12.6 ± 2.2	2.55 ± 0.40	3.47 ± 0.26	1.26 ± 0.14	13.7 ± 1.8
**C4**	8.2 ± 1.8	9.3 ± 2.7	5.92 ± 0.46	5.75 ± 0.39	6 ± 1	1.39 ± 0.26	1.81 ± 0.17	0.64 ± 0.11	22.9 ± 2.1
**C5**	< 6.3	< 8.6	< 1.3	< 1.3	< 7.5	< 2.0	< 0.8	< 0.6	12.6 ± 3.2
**V1**	33.75 ± 5.16	24.8 ± 5.2	27.35 ± 2.93	24.8 ± 1.2	26.3 ± 5.6	47 ± 2	49.6 ± 5.7	17.7 ± 1.5	539 ± 32
**V2**	139.5 ± 21.2	128 ± 23	137 ± 15	124.5 ± 5.4	17.10 ± 4.54	34.55 ± 2.09	38.30 ± 4.45	13.45 ± 1.27	78.85 ± 6.48
**V3**	24.1 ± 3.4	54.6 ± 13.3	47.88 ± 8.50	44.33 ± 7.22	44.17 ± 8.73	28.68 ± 1.79	30.45 ± 2.22	11.06 ± 6.73	338 ± 140
**V4**	29.5 ± 7.7	53 ± 12	57.75 ± 6.29	54.8 ± 2.8	51.5 ± 12	30.9 ± 1.9	32.6 ± 3.8	11.3 ± 1.1	384.5 ± 24.7
**V5**	22.85 ± 5.98	16 ± 9	14.5 ± 1.0	13.85 ± 0.95	24.75 ± 6.78	23.9 ± 2.3	26.2 ± 1.6	9.54 ± 0.68	278.5 ± 22.5
**V6**	22.5 ± 7.4	25 ± 14	16.35 ± 1.06	15.25 ± 0.81	17.15 ± 3.54	18.95 ± 1.08	24.3 ± 1.5	7.65 ± 0.52	321.5 ± 20.1
**V7**	25.10 ± 3.74	18.75 ± 6.33	21.40 ± 1.44	20.20 ± 1.08	22.20 ± 5.10	27.25 ± 1.48	27.50 ± 1.73	9.78 ± 0.71	547.50 ± 33.94
**G1**	208 ± 20	213 ± 36	213 ± 34	193 ± 10	187 ± 16	71.2 ± 5.4	79 ± 13	27.1 ± 2.3	1149 ± 98
**G2**	155 ± 20	153 ± 38	150 ± 24	138.5 ± 7.7	152 ± 18	105.4 ± 8.8	114 ± 19	39.9 ± 3.6	1090 ± 94
**G3**	133 ± 16	131 ± 31	128 ± 21	118.4 ± 6.3	118 ± 15	172 ± 13	183 ± 29	63.4 ± 5.3	1195 ± 102
**G4**	137.2 ± 9.8	129.8 ± 10.7	123.9 ± 5.9	114.2 ± 9.4	119.2 ± 11.1	105.8 ± 11.8	111.4 ± 9.7	44.4 ± 31.3	127 ± 374
**G5**	402 ± 44	264 ± 66	335 ± 28	311 ± 16	301 ± 31	41.2 ± 2.8	45.5 ± 3.8	17.2 ± 1.5	1055 ± 90
**G6**	314 ± 41	205 ± 56	252 ± 21	237 ± 12	225 ± 30	72.8 ± 4.8	78.6 ± 6.6	28.5 ± 2.5	957 ± 83
**G7**	145 ± 20	84 ± 27	102 ± 9	97.2 ± 5.1	94 ± 14	72.5 ± 4.6	76.5 ± 6.4	29.2 ± 2.5	1031 ± 88
**G8**	186 ± 24	151 ± 35	162 ± 13	152.5 ± 8.0	172 ± 22	16.3 ± 1.5	18.1 ± 1.6	6.97 ± 0.70	1105 ± 95
**G9**	171 ± 32	66 ± 38	95.3 ± 8.0	91.7 ± 5.0	50 ± 14	187 ± 12	203 ± 17	73.1 ± 6.1	935 ± 81
**G10**	159 ± 21	109 ± 30	127 ± 11	119.2 ± 6.2	125 ± 18	50.8 ± 3.3	54.7 ± 4.5	20.4 ± 1.7	944 ± 81
**G11**	198 ± 24	151 ± 36	166 ± 14	155.4 ± 8.0	156 ± 20	71.6 ± 4.5	75.2 ± 6.2	27.9 ± 2.4	1049 ± 90
**G12**	98 ± 18	56 ± 24	75.1 ± 6.3	71.7 ± 4.0	77 ± 20	49.6 ± 3.4	55.1 ± 4.6	19.9 ± 1.8	785 ± 68
**G13**	149 ± 20	109 ± 28	116 ± 10	109.9 ± 5.8	112 ± 16	68.3 ± 4.3	73.1 ± 6.1	27.1 ± 2.3	947 ± 81
**G14**	201 ± 28	170 ± 40	181 ± 15	172.3 ± 9.0	163 ± 24	33.9 ± 2.4	35.3 ± 3.0	14.3 ± 1.3	1078 ± 93
**G15**	113 ± 21	74 ± 27	72.1 ± 6.1	65 ± 4	78 ± 17	31.3 ± 2.3	34.4 ± 2.9	13 ± 1	1105 ± 95

Table S3. *Chemical composition (wt.%) of the aggregate samples used in the validation of the models.*

Sample	SiO_2	CaO	Al_2O_3	Fe_2O_3	MgO	MnO	Na_2O	K_2O	TiO_2	P_2O_5	SO_3	LoI*
**Siliceous Agg.**	96.80	0.10	1.50	0.40	0.00	0.00	0.00	0.60	0.00	0.00	0.00	0.00
**Carbonate Agg.**	0.43	30.76	0.00	0.04	22.83	0.00	0.00	0.00	0.00	0.00	0.00	45.93
**Granitic Agg. 1**	66.06	2.60	16.02	3.84	1.51	0.07	3.85	3.92	0.62	0.00	0.00	1.42
**Granitic Agg. 2**	73.03	2.28	13.00	2.08	0.62	0.04	3.41	4.59	0.28	0.00	0.00	0.65

**LoI: Lost on Ignition.*

Table S4. *Activity concentration (Bq kg^{-1}) of the aggregate samples used in the validation of the models.*

Series	^{238}U Radioactive Serie					^{232}Th Radioactive Serie			^{40}K
**Sample**	^{234}Th	^{226}Ra	^{214}Pb	^{214}Bi	^{210}Pb	^{228}Ac	^{212}Pb	^{208}Tl	
**Siliceous Agg.**	6.8 ± 3.1	11.4 ± 5.9	6.30 ± 0.62	6.41 ± 0.73	< 4.6	7.90 ± 0.79	7.7 ± 1.0	3.00 ± 0.30	167 ± 16
**Carbonate Agg.**	34.6 ± 5.2	30.7 ± 6.9	35.5 ± 3.0	33.5 ± 1.9	37.2 ± 5.1	< 1.2	< 0.6	< 0.3	< 3.2
**Granitic Agg. 1**	64.0 ± 7.1	57.5 ± 11.9	64.6 ± 3.9	60.9 ± 2.8	52.50 ± 6.14	45.85 ± 2.08	47.15 ± 2.83	17.2 ± 1.1	967.5 ± 59.0
**Granitic Agg. 2**	50.75 ± 4.94	54.0 ± 8.1	55.3 ± 3.3	51.5 ± 2.1	45.45 ± 4.32	49.50 ± 2.45	51.55 ± 3.08	18.65 ± 1.17	1.068.5 ± 64.7
